# Comparing the human milk antibody response after vaccination with four COVID-19 vaccines: A prospective, longitudinal cohort study in the Netherlands

**DOI:** 10.1016/j.eclinm.2022.101393

**Published:** 2022-04-18

**Authors:** Hannah G. Juncker, Sien J. Mulleners, Eliza J.M. Ruhé, Esmée R.M. Coenen, Sjors Bakker, Maritt van Doesburg, Jolinda E. Harinck, Romee D. Rood, Joey H. Bouhuijs, Melissa Oomen, Prof. Christianne J.M. de Groot, Prof. Dasja Pajkrt, Aniko Korosi, Prof. Johannes B. van Goudoever, Marit J. van Gils, Britt J. van Keulen

**Affiliations:** a Amsterdam UMC, Vrije Universiteit, University of Amsterdam, Emma Children's Hospital, Amsterdam Reproduction & Development Research Institute, Department of Pediatrics, Amsterdam, the Netherlands; b Swammerdam Institute for Life Sciences - Center for Neuroscience, University of Amsterdam, Amsterdam, The Netherlands; c Amsterdam UMC, University of Amsterdam, Amsterdam Infection and Immunity Institute, Department of Medical Microbiology and Infection Prevention, Amsterdam, the Netherlands; d Amsterdam UMC, Vrije Universiteit, Amsterdam Reproduction & Development Research Institute, Department of Obstetrics and Gynaecology, Amsterdam, the Netherlands

**Keywords:** COVID-19, Human milk, Antibodies, Vaccination, SARS-CoV-2

## Abstract

**Background:**

Vaccination of lactating women against COVID-19 may protect not only themselves but also their breastfed infant through human milk. Therefore, it is important to gain insight into the human milk antibody response after immunization with the various vaccines that are currently widely used. The aim of this study is to determine and compare the antibody response in human milk following vaccination with mRNA- and vector-based vaccines up to over two months post-vaccination.

**Methods:**

This prospective cohort study was conducted in the Netherlands between January 06, 2021 and July 31, 2021. Participants were recruited through social media. Human milk samples were collected longitudinally during a period of 70 days from women receiving one of the four different severe acute respiratory coronavirus 2 (SARS-CoV-2) vaccines: Pfizer-BioNTech (BNT162b2), Moderna (mRNA-1273), Oxford/AstraZeneca (AZD1222) and Johnson&Johnson (Ad26.COV2.S). SARS-CoV-2-specific antibodies were measured using an enzyme-linked immunosorbent assay. The area under the curve (AUC) of the Immunoglobulins A (IgA) and G (IgG) antibody response was determined over 15 and 70 days following the first vaccination and compared between the different vaccines.

**Findings:**

This study enrolled 134 vaccinated lactating women of whom 97 participated the entire study period. In total, 1887 human milk samples were provided. The human milk antibody response differed between SARS-CoV-2 vaccines over the study period. The mean AUC of SARS-CoV-2-specific IgA, but not IgG, in human milk over 15 days was higher after vaccination with an mRNA-based vaccine than a vector-based vaccine (AUC with respect to ground [AUCg] ± the standard error of the mean [SEM] for IgA was 6·09 ± 0·89 in the BNT162b2 group, 7·48 ± 1·03 in the mRNA-1273 group, 4·17 ± 0·73 in the AZD1222 group, and 5·71 ± 0·70 in the Ad26.COV2.S group). Over a period of 70 days, the mean AUCg of both IgA and IgG was higher after vaccination with an mRNA-based vaccine than a vector-based vaccine (AUCg ± SEM for IgA was 38·77 ± 6·51 in the BNT162b2 group, 50·13 ± 7·41 in the mRNA-1273 group, 24·12 ± 5·47 in the AZD1222 group, and 28·15 ± 6·69 in the Ad26.COV2.S group; AUCg ± SEM for IgG was 40·43 ± 2·67 in the BNT162b2 group, 37·01 ± 2·38 in the mRNA-1273 group, 16·04 ± 5·09 in the AZD1222 group, and 10·44 ± 2·50 in the Ad26.COV2.S group).

**Interpretation:**

Overall, maternal vaccination during lactation with an mRNA-based vaccine resulted in higher SARS-CoV-2 antibody responses in human milk compared to vector-based vaccines. Therefore, vaccination with mRNA-based vaccines, preferably with the mRNA-1273 vaccine, might not only provide better immunological protection for the mother but also for her breastfed infant**.**

**Funding:**

Stichting Steun Emma Kinderziekenhuis and the Amsterdam Infection and Immunity Institute (grant 24175).

**Research in context**

**Evidence before this study**

Before starting this study (December 2020), PubMed and MedRxiv were searched for articles on the human milk antibody response after severe acute respiratory coronavirus 2 (SARS-CoV-2) vaccination during lactation, with the terms: SARS-CoV-2/COVID-19, vaccin*, human milk, antibodies or synonyms. No studies that investigated the antibody response in human milk after vaccination with a SARS-CoV-2 vaccine were identified. Repeating this search before data interpretation (from database inception up to October 31, 2021), we only identified published studies that focussed on the antibody response in human milk after vaccination with mRNA-based vaccines. One preprinted study was identified on the effect of vaccination with a vector-based vaccine. These studies were hampered by their small sample sizes and relatively short follow-up times.

**Added value of this study**

Knowledge on differences in the human milk antibody responses after mRNA-based and vector-based vaccines is limited. Vaccination during lactation may not only protect the mother, but also her breastfed infant through human milk. Therefore, the results of this study on the human milk antibody response after different SARS-CoV-2 vaccines are important to guide health care workers and lactating mothers in their decision regarding vaccination against COVID-19.

**Implications of all the available evidence**

Based on our findings, we suggest that an mRNA-based vaccine, preferably mRNA-1273, is the best choice for lactating mothers, as it might provide the best immunological protection for their breastfed infant.

Alt-text: Unlabelled box

## Introduction

In children, coronavirus disease 2019 (COVID-19) usually has a mild course. However, newborns and infants are more susceptible to a severe disease course.^1^, ^2^, ^3^, ^4^, ^5^ Almost 20% of hospitalized children with COVID-19 are younger than 3 months old.^6^ The vulnerability of infants is partially due to an immature immune system, and therefore the infant relies on passive immunity derived from its mother through the placenta and via human milk.^7^^,^^8^ Human milk is suggested to play an important role in the protection against infectious diseases, which are lower in breastfed compared to formula-fed infants.^9^^,^^10^ Human milk enhances the infant's immune system as it contains bioactive components, including immunoglobulins, oligosaccharides, lactoferrin, and other mediators with pro- and anti-inflammatory factors.^11^ The dominant antibody in human milk is secretory immunoglobulin A (IgA).^8^ IgA has an important anti-inflammatory function by prohibiting the binding of microbes to the mucosal membranes of infants and neutralizing microbial toxins.^10^, ^11^, ^12^ In addition, a protective role of human milk immunoglobulin G (IgG) was suggested recently.^13^

Antibodies against the virus are present in human milk of mothers who have previously been infected with the severe acute respiratory coronavirus 2 (SARS-CoV-2).^12^^,^^14^, ^15^, ^16^ These specific antibodies have neutralizing capacity and are suggested to provide immunity to infants.^14^^,^^15^^,^^17^ The advantages of breastfeeding and the absence of vertical transmission of SARS-CoV-2 via human milk^14^^,^^18^, ^19^, ^20^, ^21^ have led to the advice of the World Health Organization to encourage mothers to continue breastfeeding their infant during the COVID-19 pandemic.^22^

Vaccination strategies in many countries worldwide report on a strong reduction of severe COVID-19 cases in adults. However, lactating women have been excluded due to safety concerns. As such, vaccination against COVID-19 has been discouraged for a long time in this specific group.^23^ This resulted in a lack of knowledge on the effect of vaccination in lactating women and its effects on human milk. To date, only small sample sized studies with short follow-up time have been conducted on the effect of maternal SARS-CoV-2 vaccinations during lactation and showed the presence of SARS-CoV-2-specific antibodies in human milk after vaccination with mRNA-based vaccines,^24^, ^25^, ^26^, ^27^, ^28^, ^29^, ^30^, ^31^ while the effect of vector-based vaccines on SARS-CoV-2-specific antibodies in human milk is yet unknown.

The current study aims to 1) determine the antibody response in human milk following vaccination over a period of 70 days; and 2) compare antibody responses after vaccination with: the BNT162b2 vaccine, or the mRNA-1273 vaccine (both mRNA-based vaccines), or the AZD1222 vaccine, or the Ad26.COV2.S vaccine (both vector-based vaccines). Based on the fact that mRNA-based vaccines' efficacy is higher than the vector-based vaccines^23^ and the positive correlation between the efficacy of vaccines and the amount of antibodies,^32^^,^^33^ we hypothesize that mRNA-based vaccines will generate a higher human milk antibody response compared to vector-based vaccines.

## Methods

### Study design

This prospective longitudinal cohort study is a follow-up study of the COVID MILK – POWER MILK study.^34^ All samples were subjected to longitudinal analysis of specific antibodies against SARS-CoV-2 in human milk and serum after vaccination against COVID-19 with either BNT162b2/Comirnaty developed by Pfizer-BioNTech, mRNA-1273/Spikevax developed by Moderna, AZD1222/Vaxzevria developed by Oxford/AstraZeneca and Ad26.COV2.S developed by J&J/Janssen. Ethical approval was acquired from the Independent Ethics Committee of the Vrije Universiteit Medical Center (2020.425/NL74752.029.20). The study was conducted in accordance with the principles of the declaration of Helsinki and the ICH GCP Guidelines, and the Regulation on Medical Research involving Human subjects and reported in adherence to the CONSORT reporting guidelines.

### Setting

We conducted this single-center initiated study throughout the Netherlands, where the SARS-CoV-2 vaccination program started on January 06, 2021. Data collection was performed between January 06, 2021 and July 31, 2021. Human milk samples were collected longitudinally over a period of 70 days (Supplemental Figure 1). In total, up to 17 samples of human milk were collected per lactating woman according to the following schedule: one sample before the first vaccination and one sample on days 3, 5, 7, 9, 11, 13, and 15 to 17 after the first vaccination. This schedule was the same for the second vaccination, except for Ad26.COV2.S as these participants only receive one dose. The last sample was collected 56-84 days after the first vaccination date, except for the AZD1222 group who collected the last sample around day 98. To minimize bias, participants were instructed to empty one breast in the morning, before the first feeding moment, and collect 5 ml of milk after mixing the milk. Participants were requested to store the milk samples in the freezer. Four blood samples were collected to determine the levels of circulating immunoglobulin G (IgG); before each dose of the vaccine and 15–17 days after both doses and not at the last time point.

### Participants

Lactating women in the Netherlands receiving one of the above-described SARS-CoV-2 vaccines were eligible to participate. Participants were recruited through several social media platforms. There were no exclusion criteria. Due to logistical reasons, the study aimed to include a minimum of 20 women per vaccine group. All participants were requested to send their vaccination certificate, including the type of vaccination and lot number. Written informed consent was obtained from all participants.

### Measurements

Before analysis, the collected human milk and serum samples were stored at the Amsterdam University Medical Centre, location Vrije Universiteit Medical Centre (VUmc), at -80°C. To assess the SARS-CoV-2-specific IgA and IgG antibodies in human milk and IgG in serum, an enzyme-linked immunosorbent assay with the SARS-CoV-2 spike protein was used as described previously.^17^ Samples were considered positive at an OD450nm value of 0·5 for IgA in human milk, 0·2 for IgG in human milk and 0·3 for IgG in the serum samples. With these cutoff values, for IgA in human milk the sensitivity was 67·9% (95% CI: 61·0–74·1%) and the specificity was 99·0% (95% CI: 94·7–100·0%), for IgG in human milk the sensitivity was 96·3% (95% CI: 89·6–99·0%) and the specificity 98·8% (95% CI: 93·4–99·9%). For serum IgG, the sensitivity was 99·2% (95% CI: 97·1–99·9%) with a specificity of 94·8% (95% CI: 87·2–97·9%).

### Statistical analysis

For the maternal and infant descriptive statistics, IBM SPSS Statistics for Windows (Version 26) was used. Discrete variables are presented as the number and percentage of observations within a group, and continuous variables are presented as the mean with standard deviation (SD) or the median with interquartile range (IQR), depending on the distribution. Possible confounding factors were compared between groups. To determine differences in group characteristics, the Chi-square test, Kruskal Wallis test, or one-way-ANOVA test were used as appropriate.

The different dynamics of IgA levels in human milk and IgG levels in serum after vaccination are displayed using Graphpad Prism 9.1.0 for Windows. The variability of human milk IgA levels between the groups is represented by the coefficient of variation, abbreviated by CV. To assess the total longitudinal antibody response, we determined the area under the curve with respect to ground (AUCg), with respect to increase (AUCi), and the area under the curve with respect to the cut-off, both the area of positive peaks (AUC_cutoffpp_) and the net area which subtracts the area of peaks below the cut-off (AUC_cutoffnet_), as described by Pruessner, Kirschbaum, Meinlschmid and Hellhammer.^35^ AUCs were calculated with actual data, there was no data imputation. The AUCg measures the entire two-dimensional area under the mean antibody line. This area represents the total antibody response after vaccination and also includes measurements below the cut-off value, which is important as the sensitivity of human milk antibodies was 67·9%. The AUCi is calculated with reference to the first antibody measurement and thereby emphasizes the changes over time. The AUC_cutoffpp_ measures the two-dimensional area under the mean antibody line until the cut-off. This area represents only the detectable antibody response as defined above, and hereby it is not biased by background signals. The AUC_cutoffnet_ is the difference computed by subtracting the area below the cut-off from the area above the cut-off. Thus, in contrast to the AUC_cutoffpp,_ this area also takes into account the areas below the cut-off. These AUCs were calculated for the initial response, up to 15 days after the first dose, and for a total period of 70 days. As the second dose of the AZD1222 vaccine was not within the time window of 70 days, these data points were shifted to the time point where the last data point of the AZD1222 vaccine coincided with 70 days to be able to include these data points in the AUC analyses. The mean total AUC with standard error of the mean (SEM) and degrees of freedom (df) for the study groups were compared using a one-way ANOVA. A p-value of ≤0*·*05 was considered as statistically significant, we did not correct for multiple testing. GraphPad Prism version 9.1.0 for Windows was used to determine and compare the AUCs for both groups.^36^

### Role of Funding sources

The funders of the study had no role in study design, data collection, data analysis, data interpretation, or writing of the report.

## Results

### Study population

**Figure 1:**
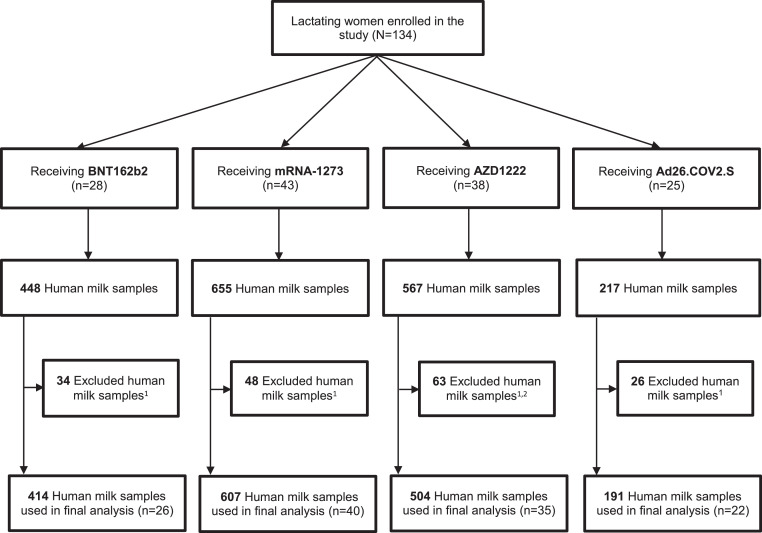
Flowchart of participants. ^1^ These samples have been excluded because of positive result for SARS-CoV-2-specific IgA in human milk before vaccination (day 0). ^2^ Twenty-four of the 63 samples have been excluded because the participants received a heterologous vaccine schedule due to changes in the vaccination policy.

In total, 1887 human milk samples and 412 serum samples were collected (Figure 1). Participants (n=11) who had detectable SARS-CoV-2-specific antibodies at baseline were excluded from further analyses. Eventually, a total amount of 1716 human milk samples were analyzed for human milk antibodies. The characteristics of the participants and their infants are presented in Table 1. The median duration of breastfeeding was between 4·5 to 7·5 months and was the highest in the BNT162b2 group. Study groups did not differ in pre-existing conditions. None of the participants used immunosuppressive medication.

**Table 1:** Characteristics of participants and their infants at baseline.

	No. (%) of patients per vaccination group				
Characteristic	BNT162b2 (n=28)	mRNA-1273 (n=43)^a^	AZD1222 (n=38)ᵃ	Ad26.COV2.S (n=25)	*P-value*
Mother					
Age mother (years) – median (IQR)	34·0 (4)	33·5 (6)	33·0 (4)	32·0 (5)	0·42
BMI (kg-m²)^b^ – median (IQR)	24·0 (5·6)	22·9 (5·5)	23·1 (5·0)	23·4 (3·2)	0·89
Level of education^c^					0·19
Low	0 (0·0)	0 (0·0)	0 (0·0)	0 (0·0)	-
Middle	3 (10·7)	4 (10·0)	6 (17·1)	25 (100)	-
High	25 (89·3)	36 (90)	29 (82·9)	0 (0·0)	-
Vaccination degree					
Child-immunization^d^	28 (100)	40 (100)	34 (97·1)	25 (100)	0·44
Pertussis vaccination during pregnancy	27 (96·4)	38 (95·0)	34 (97·1)	24 (96·0)	0·97
Other^e^	15 (53·6)	25 (62·5)	22 (62·9)	17 (68·0)	0·75
Prior PCR-proven SARS-CoV-2 infection	1 (3·6)	2 (5·0)	2 (5·7)	2 (8·0)	0·91
Chronic disease	3 (10·7)	8 (20·0)	7 (20·0)	4 (16·0)	0·74
Autoimmune disease^f^	2 (7·1)	5 (12·5)	0 (0·0)	1 (4·0)	0·15
Immunosuppressive medication	0 (0·0)	0 (0·0)	0 (0·0)	0 (0·0)	-
Parity primiparous	13 (46·4)	13 (32·5)	16 (45·7)	13 (52·0)	0·41
Vaginal delivery	20 (71·4)	31 (77·5)	27 (77·1)	19 (76·0)	0·94
Lactation stage (months)^g^ - median (IQR)	7·5 (4·5)	5·2 (4·0)	5·2 (5·0)	4·5 (4·2)	***0·028***
Infant					
Gestational age (weeks) - median (IQR)	39·3 (1·8)	40·3 (1·8)	40·4 (2·4)	40·3 (1·9)	0·27
Birth weight (grams) – median (IQR)	3400 (520)	3619 (538)	3608 (674)	3722 (749)	0·39
Exclusively breastfeeding	13 (46·4)	19 (47·5)	18 (51·4)	14 (56·0)	0·89

Abbreviations: No., number; IQR, interquartile range.

a Characteristics of 3 participants were missing because of no completion of the questionnaire.

b BMI: Body Mass Index, calculated by participant's weight in kilograms divided by the square of the participant's height in centimeters.

c Level of education conformed to International Standard Classification of Education 2011.

d According to the Dutch National Immunization Program.

e Other vaccinations specified: hepatitis A and B, yellow fever, rabies, typhoid fever, influenza A, Bacille Calmette Guerin (BCG), meningitis C, and cholera

f Auto-immune disorder: hypothyroidism (n=4), diabetes mellitus type 1 (n = 1), Graves (n = 1), SLE like disease (n = 1), Coeliac (n =1).

g The lactation stage was defined as the period between an infant's date of birth and the date of the first human milk collection in months. The lactation stage of the BNT162b2 group was higher compared to all other groups.

### SARS-CoV-2-specific IgA in human milk

#### Individual SARS-CoV-2-specific IgA in human milk

The individual antibody responses are displayed in Supplemental Figure 2. Individual antibody responses were variable, with a higher CV after vaccination with mRNA-based vaccines (38·3% and 43·2% after BNT162b2 and mRNA-1273, respectively) compared to the vector-based vaccines (18·8% and 20·9% after AZD1222 and Ad26.COV2.S respectively) (p<0·0001).

Overall, the rise in antibody levels is higher after vaccination with the mRNA-based vaccines. Fifteen days after vaccination, more participants showed antibodies above the cut-off after vaccination with an mRNA-based vaccine than a vector-based vaccine (76·0%, 88·2%, 20·0%, 45·5% after BNT162b2, mRNA-1273, AZD1222 and Ad26.COV2.S, respectively). Similarly, after the second dose, detectable antibodies were observed more frequently in the mRNA groups. Over the whole study period, 4 and 3% of the participants did not show detectable antibodies after vaccination with BNT162b2 and mRNA-1273, respectively, whereas 63% and 50% did not show antibody levels above the cut-off after vaccination with AZD1222 and Ad26.COV2.S, respectively.

#### Mean SARS-CoV-2-specific IgA in human milk

**Figure 2:**
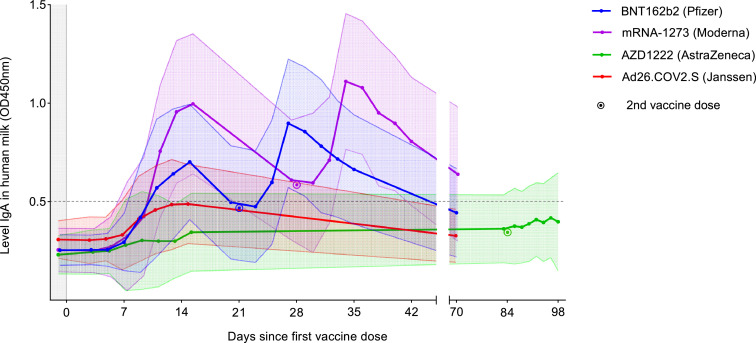
Mean levels of SARS-CoV-2-specific Immunoglobulin A (IgA) in human milk of all groups. The dotted line indicates the cut-off value for level SARS-CoV-2 specific IgA in human milk; filled area between error lines indicates the standard deviation.

Figure 2 illustrates the mean levels of SARS-CoV-2-specific IgA in human milk of all vaccine groups, in which all human milk samples with a positive IgA level before the first vaccination have been excluded. A noticeable biphasic course was observed after vaccination with an mRNA-based vaccine, while participants receiving a vector-based vaccine on average did not show milk conversion (human milk antibody levels above the cut-off) during the study period. The human milk SARS-CoV-2-specific IgA started to rise on days 3 to 5 after vaccination in all groups, reaching a peak on day 15. The highest peak at 15 days after the first vaccine was observed after vaccination with the mRNA-1273 vaccine, which was 1·4, 2·9, and 2·0 times higher compared to BNT162b2, AZD1222 and Ad26.COV2.S, respectively (*p<0·0001*).

After the second dose, an accelerated and higher immune response was observed for all three vaccines. After 2 months, the mean human milk IgA levels of the BNT162b2 and the mRNA-1273 group were significantly elevated compared to the levels before vaccination (*p=0·0003, p<0·0001* respectively), whereas only the mRNA-1273 group retained milk conversion after 2 months. The Ad26.COV2.S group showed no significantly increased level of IgA 2 months after vaccination compared to pre-vaccination levels (*p=0·61*). Fifteen days after the second dose of AZD1222, the mean IgA level was also significantly higher than the pre-vaccination level (*p=0·0007*), but on average, no milk conversion was reached.

#### Area under the curve for IgA in human milk

The calculated AUC_cutoffpp,_ AUC_cutoffnet,_ AUC_G,_ and the AUC_I_ are shown in Table 2a. Over the first 15 days after vaccination, all AUCs differed between study groups. Over a period of 70 days, AUC_cutoffnet,_ AUC_G,_ and the AUC_I_ also differed between groups.

**Table 2a:** Area under the curve of SARS-CoV-2-specific IgA in human milk with respect to the cut-off (positive peaks and net area), with respect to the ground, and with respect to the increase, 15 days and 70 days after vaccination.

	BNT162b2 (n=26)	mRNA-1273 (n=40)	AZD1222 (n=35)	Ad26.COV2.S (n=22)	*P-value*^a^
**Period of first 15 days**					
df^b^	199	257	267	167	
AUC_cutoffpp_	0·58 ± 0·67	1·85 ± 0·75	0·00 ± 0·00	0·00 ± 0·00	***0·031***
AUC_cutoffnet_	-1·44 ± 0·80	-0·05 ± 0·96	-3·36 ± 0·73	-1·82 ± 0·70	***0·026***
AUC_G_	6·09 ± 0·89	7·48 ± 1·03	4·17 ± 0·73	5·71 ± 0·70	***0·039***
AUC_I_	2·28 ± 0·89	3·66 ± 1·02	0·71 ± 0·73	1·10 ± 0·69	***0·050***
**Period of 70 days**					
df	397	590	488	182	
AUC_cutoffpp_	5·97 ± 3·85	16·90 ± 7·38	0·00 ± 0·00	0·00 ± 0·00	0·070
AUC_cutoffnet_	3·95 ± 3·87	14·99 ± 7·40	-11·02 ± 5·47	-6·99 ± 6·69	***0·012***
AUC_G_	38·77 ± 6·51	50·13 ± 7·41	24·12 ± 5·47	28·15 ± 6·69	***0·023***
AUC_I_	20·99 ± 6·51	32·28 ± 7·41	7·96 ± 5·47	6·61 ± 6·69	***0·026***

Values displayed are the mean area under the curves ± standard error of the mean.

Abbreviations: df, degrees of freedom; AUC, area under the curve; AUC_cutoffpp_, positive peaks above cut-off; AUC_cutoffnet_, net area with respect to cut-off; AUC_G_, with respect to ground; AUC_I_, with respect to increase.

a AUC values were compared between vaccine groups using an one-way ANOVA test, statistical significance was determined (p≤0*·*05). ^b^ based on the number of samples with an enzyme-linked immunosorbent assay result.

### SARS-CoV-2-specific IgG in human milk

**Figure 3:**
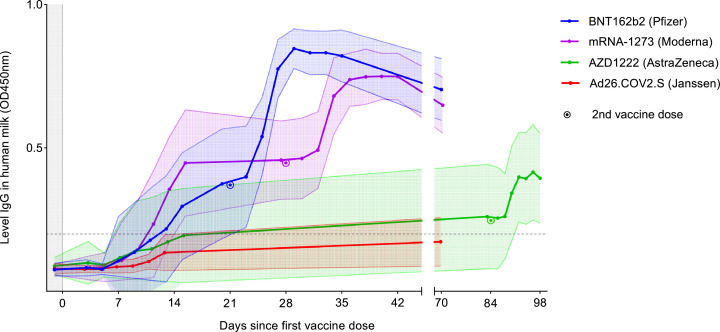
Mean levels of SARS-CoV-2-specific Immunoglobulin G (IgG) in human milk of all groups. The dotted line indicates the cut-off value for level SARS-CoV-2 specific IgG in human milk; filled area between error lines indicates the standard deviation.

The mean levels of SARS-CoV-2-specific IgG in human milk of all vaccine groups are displayed in Figure 3. All human milk samples with a positive IgG level before the first vaccination have been excluded from Figure 3. The human milk SARS-CoV-2-specific IgG started to rise on day 5 to 7 after vaccination in all groups and gradually increased in the first 15 days after vaccination, with the highest value in the mRNA-1273 group, which was 1·5, 2·3, and 2·6 times higher compared to the BNT162b2, AZD1222, and Ad26.COV2.S groups, respectively (p<0·0001). In contrast to the dynamics of human milk IgA, human milk IgG did not decrease before the second dose of the vaccine. After the second dose, an accelerated and higher immune response was observed for all three vaccines, comparable to our findings in human milk IgA. The groups of BNT162b2, mRNA-1273, and AZD1222 still showed detectable IgG antibodies in human milk 70 days after the first dose of the vaccine, whereas only the mRNA-1273 group showed detectable IgA levels. For the Ad26.COV2.S group no milk conversion was reached during the study period on average. The CV was higher in the mRNA-based vaccines (BNT162b2 72·5%, mRNA-1273 61·7%) than the vector-based vaccine of Ad26.COV2.S 33·7% *(p=0·0016).*

#### Area under the curve for IgG in human milk

The calculated AUC_cutoffpp_, AUC_cutoffnet_, AUC_G_, and the AUC_I_ are shown in Table 2b. Over the first 15 days after vaccination, AUCs did not differ between study groups. Over a period of 70 days, all AUCs differed between groups, with the highest values for the mRNA-based vaccines (p<0·0001).

**Table 2b:** Area under the curve of SARS-CoV-2-specific IgG in human milk with respect to the cut-off (positive peaks and net area), with respect to the ground, and with respect to the increase, 15 days and 70 days after vaccination.

	BNT162b2 (n=26)	mRNA-1273 (n=40)	AZD1222 (n=35)	Ad26.COV2.S (n=22)	*P-value*
**Period of first 15 days**					
df	195	266	284	175	
AUC_cutoffpp_	0·12 ± 0·28	0·61 ± 0·38	0·00 ± 0·00	0·00 ± 0·00	0·21
AUC_cutoffnet_	-0·95 ± 0·48	-0·38 ± 0·48	-1·09 ± 0·52	-1·53 ± 0·14	0·40
AUC_G_	2·05 ± 0·53	2·62 ± 0·53	1·91 ± 0·52	1·47 ± 0·14	0·45
AUC_I_	0·91 ± 0·53	1·47 ± 0·53	0·59 ± 0·52	0·29 ± 0·14	0·37
**Period of 70 days**					
df	393	607	520	191	
AUC_cutoffpp_	27·50 ± 2·63	24·00 ± 2·35	3·13 ± 3·50	0·00 ± 0·00	***<0·0001***
AUC_cutoffnet_	26·43 ± 2·66	23·01 ± 2·37	2·04 ± 3·54	-3·56 ± 2·50	***<0·0001***
AUC_G_	40·43 ± 2·67	37·01 ± 2·38	16·04 ± 5·09	10·44 ± 2·50	***<0·0001***
AUC_I_	35·13 ± 2·67	31·67 ± 2·38	9·86 ± 5·09	4·95 ± 2·50	***<0·0001***

Values displayed are the mean area under the curves ± standard error of the mean

Abbreviations of table are explained in the footnote of Table 2a.

### SARS-CoV-2 specific IgG in serum

#### Levels of SARS-CoV-2-specific IgG in serum

Supplemental Figure 3 illustrates the mean levels of SARS-CoV-2-specific IgG in serum of all groups over the study period. The serum samples of ten participants have been excluded due to seroconversion at baseline. Fifteen days after the first and fifteen days after the second vaccination, SARS-CoV-2-specific IgG antibody levels of the study groups differed from each other (p<0·0001).

## Discussion

In line with our hypothesis, we demonstrated not only that more participants showed milk conversion after vaccination with mRNA-based vaccines,^37^ but that the SARS-CoV-2-specific antibody levels in human milk are also higher. In fact, after vaccination with vector-based vaccines, IgA levels were not detectable in human milk on a group level, and IgG levels in human milk only became detectable after vaccination with the AZD1222 vaccine. This indicates that protection in infants against COVID-19 is thus most likely through maternal vaccination with an mRNA-based vaccine. Although undeniable evidence that human milk antibodies directly protect against respiratory infections is lacking, it is very likely that these antibodies play a crucial role and that IgA provides the first line of defense.^38^^,^^39^ *In vitro*, SARS-CoV-2-specific human milk antibodies indeed have neutralizing capacity.^14^^,^^15^^,^^17^^,^^20^ Moreover, vaccination against other infectious diseases resulted in measurable antibody levels in human milk and fewer episodes of illness in the breastfed infant.^40^, ^41^, ^42^

We found that SARS-CoV-2-specific IgA has a biphasic antibody response, with an accelerated response after the second dose. After 70 days, mean IgA levels remained only detectable after vaccination with mRNA-1273. Mean human milk IgG antibodies showed a different pattern after vaccination with still detectable antibody levels at 70 days of follow-up in three out of four vaccines. Up to date, the presence and dynamics of SARS-CoV-2-specific antibodies in human milk after vaccination were only described for mRNA-based vaccines. Overall, previous studies also showed elevated human milk SARS-CoV-2-specific antibodies after vaccination. However, these studies had different study protocols in terms of sampling intervals and follow-up time, making it hard to compare details of the dynamics of human milk IgA and IgG responses with our results.^24^^,^^25^^,^^28^^,^^29^^,^^43^^,^^44^ The faster increase in IgA levels and the more stable IgG levels after vaccination that we found in human milk were described previously in serum.^45^ The different dynamics in IgA and IgG antibody responses can be explained by their different functions. Where IgA plays a key role in the initial immune response as the first line of defense against the virus, IgG is predominantly important in the secondary immune response.^46^ Dynamics in antibody levels in human milk and serum correlate as antibodies in human milk are mostly produced by plasma cells in the mammary gland, which are a direct reflection of the antigen exposure in the systemic maternal immune system.^8^ The more rapid increase in antibodies after the second dose is in line with the immunological memory that will lead to a faster response with higher antibody levels the second time the body is exposed to the antigen.^47^ This phenomenon is also illustrated by some of the individuals in our cohort who have been infected with SARS-CoV-2 in the past before vaccination (Supplemental Figure 2).

Knowledge of the antibody responses after the different vaccines is important to empower health care workers and lactating women in decision-making regarding SARS-CoV-2 vaccination in this specific group. In this study, we demonstrated differences in the human milk antibody response between different SARS-CoV-2 vaccines. Post-hoc analyses (data not shown) revealed that the differences in the IgA responses were explained by a statistical difference between the vaccines of mRNA-1273 and AZD1222. The other groups did not statistically differ from each other. The differences in the IgG responses were explained by differences between the mRNA-based vaccines and the vectors-based vaccines. The IgG responses after vaccination with mRNA-1273 or BNT162b2 did not differ from each other, neither did the IgG antibody responses after vaccination with AZD1222 or Ad26.COV2.S. Previous literature on this topic is scarce. Three studies compared human milk antibody responses after different mRNA-based vaccines of which two preprinted studies found no difference and one study found the highest human milk antibody response after vaccination with mRNA-1273.^27^^,^^28^^,^^48^ Only one study compared the antibody response between mRNA-based and vector-based vaccines, specifically BNT162b2, mRNA-1273, and AZD1222.^48^ In line with our results, they found the highest IgA and IgG responses after vaccination with the mRNA-based vaccines.

This study is strengthened by its relatively long follow-up time up to 98 days, large sample size and small sampling intervals, enabling us to create a detailed overview of the IgA and IgG antibody dynamics over time. In addition, samples were collected in a standardized way, which is important as antibody concentrations in human milk depend on the time of the day and between fore- and hindmilk. One of the limitations of this study is that we did not measure neutralizing capacity. Another limitation is that the timing of sample collection differed between study groups, specifically after the second vaccine dose, which could have influenced the results. Moreover, between 15 days after the first dose for Ad26.COV2.S or 15 days after the second dose for the other vaccines, and the follow-up moment at 70 days, no samples were collected, providing a less detailed overview of the dynamics of antibody levels over this period. Furthermore, participants in the BNT162b2 group were breastfeeding for a longer period compared to the other groups. As previous studies did not find any difference in antibody levels between lactation stages of 4 to 7 months, we did not adjust for this difference.^49^ In addition, our participants were breastfeeding for a longer time than the average lactating mother in the Netherlands, therefore our data might not be completely translatable to the total population of lactating women in the Netherlands.^50^ It is striking that all 25 women who received the Ad26.COV2.S vaccine were classified as having a middle levels of education. This could possibly be due to the fact that the mRNA-based vaccines were mostly used to vaccinate health-care workers at the beginning of the vaccination campaign. Lastly, possible risks for infants following maternal vaccination against COVID-19 were not investigated in our study. However, earlier research described no serious adverse reactions nor side effects in infants after maternal vaccination with one of the vaccines.^24^^,^^48^^,^^51^ For mRNA vaccines, the transfer of the vaccine lipid or mRNA to human milk is unlikely.^52^^,^^53^ Moreover, the genetically modified adenovirus which is used in the vector-based vaccines is made incompetent, which makes it unlikely that these vaccines cause adverse effects in the infant.^54^

As vaccination during lactation may protect the mother and her breastfed infant, knowledge on the effect of maternal vaccination is crucial to guide health care workers and lactating mothers in decision-making regarding vaccination against COVID-19. In this study, we demonstrated that the human milk antibody response differed between SARS-CoV-2 vaccines over a period of 70 days. Protection against COVID-19 through antibodies in human milk is most likely through vaccination with an mRNA-based vaccine, preferably mRNA-1273.

## Contributors

Conceptualisation: HGJ, CJMdG, DP, AK, MJvG, JBvG, BJvK. Data curation: HGJ, SJM, ERMC, MJvG, BJvK. Formal analysis: HGJ, SJM, MJvG, BJvK. Funding acquisition: MJvG, JBvG, BJvK. Investigation: HGJ, SJM, ERMC, SB, MvD, JEH, RDR, JHB, MO, BJvK. Methodology: HGJ, SJM, MJvG, JBvG, BJvK. Project administration: HGJ, SJM, EJMR, ERMC, SB, MvD, JEH, RDR, JBvG, BJvK. Resources: HGJ, MJvG, JBvG, BJvK. Supervision: JBvG, JBvK. Validation: HGJ, SJM, MJvG, JBvG, BJvK. Visualisation: HGJ, SJM, BJvK. Writing - original draft: HGJ, SJM, BJvK. Writing - review and editing: EJMR, ERMC, SB, MvD, JEH, RDR, JHB, MO, CJMdG, DP, AK, MJvG, JBvG, BJvK. HGJ, SJM, ERMC, MJvG and BJvK had access to the dataset and JBvG and BJvK had final responsibility for the decision to submit for publication. HGJ and BJvK verified the underlying study data.

## Data sharing statement

Data will be available to researchers who provide a methodologically correct proposal to achieve the aims in the approved proposal. Proposals should be directed to the corresponding author to gain access to the data. Data requestors need to sign an access agreement.

## Funding

Stichting Steun Emma Kinderziekenhuis and the Amsterdam Infection and Immunity Institute (grant 24175).

## Declaration of interests

The authors declare no competing interests.

## Appendix Supplementary materials

Image, application 1
