# Supplementary material for: Comparing the human milk antibody response after vaccination with four COVID-19 vaccines: A prospective, longitudinal cohort study in the Netherlands
Source: eClinicalMedicine. 2022 Apr 18;47:101393. doi: 10.1016/j.eclinm.2022.101393 (PMC9013951; doi:10.1016/j.eclinm.2022.101393)
Supplement: Supplementary file 1 [file mmc1.pdf]

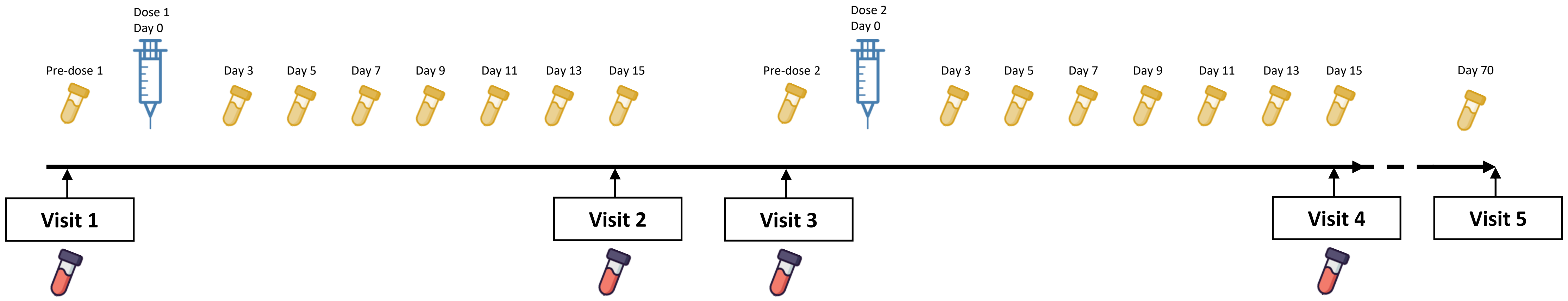

Supplemental Figure 1. Data collection.
This figure presents how and when the data was collected, depending on the dates participants received their vaccinations. The last time point, day 70, is the average visit 5 timing across all vaccine groups.

The yellow tubes indicate the day human milk samples were collected; red tubes indicate the days a vena puncture was performed to collect a serum sample; blue needles indicate vaccination date.

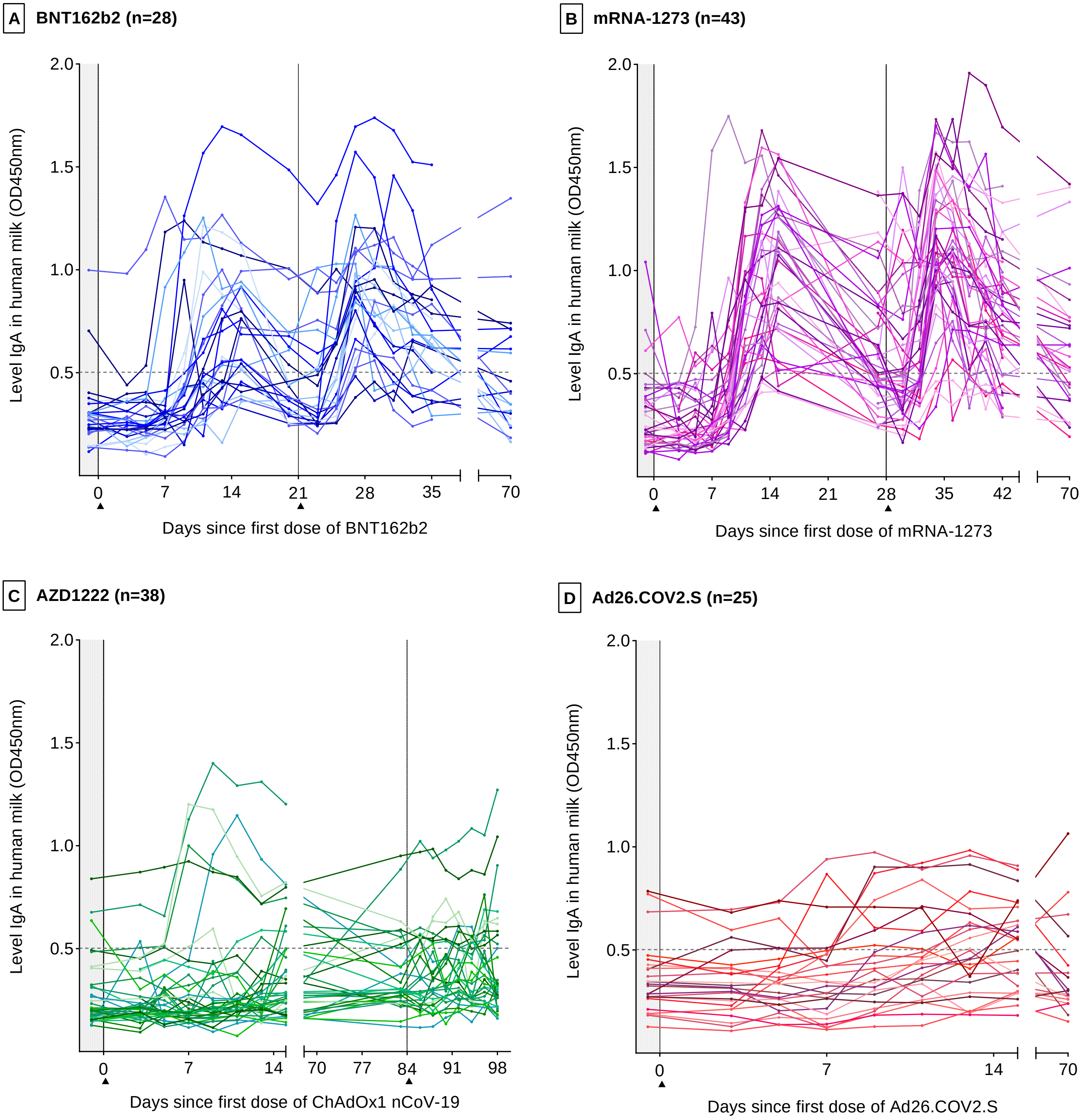

Supplemental Figure 2. Individual Immunoglobulin A (IgA) antibody responses.
Horizontal dotted line indicates cut-off value; Arrowheads, date receiving a vaccine dose
A. Individual level IgA in human milk of 28 lactating women receiving BNT162b2
B. Individual level IgA in human milk of 43 lactating women receiving mRNA-1273
C. Individual level IgA in human milk of 38 lactating women receiving AZD1222
D. Individual level IgA in human milk of 25 lactating women receiving Ad26.COV2.S

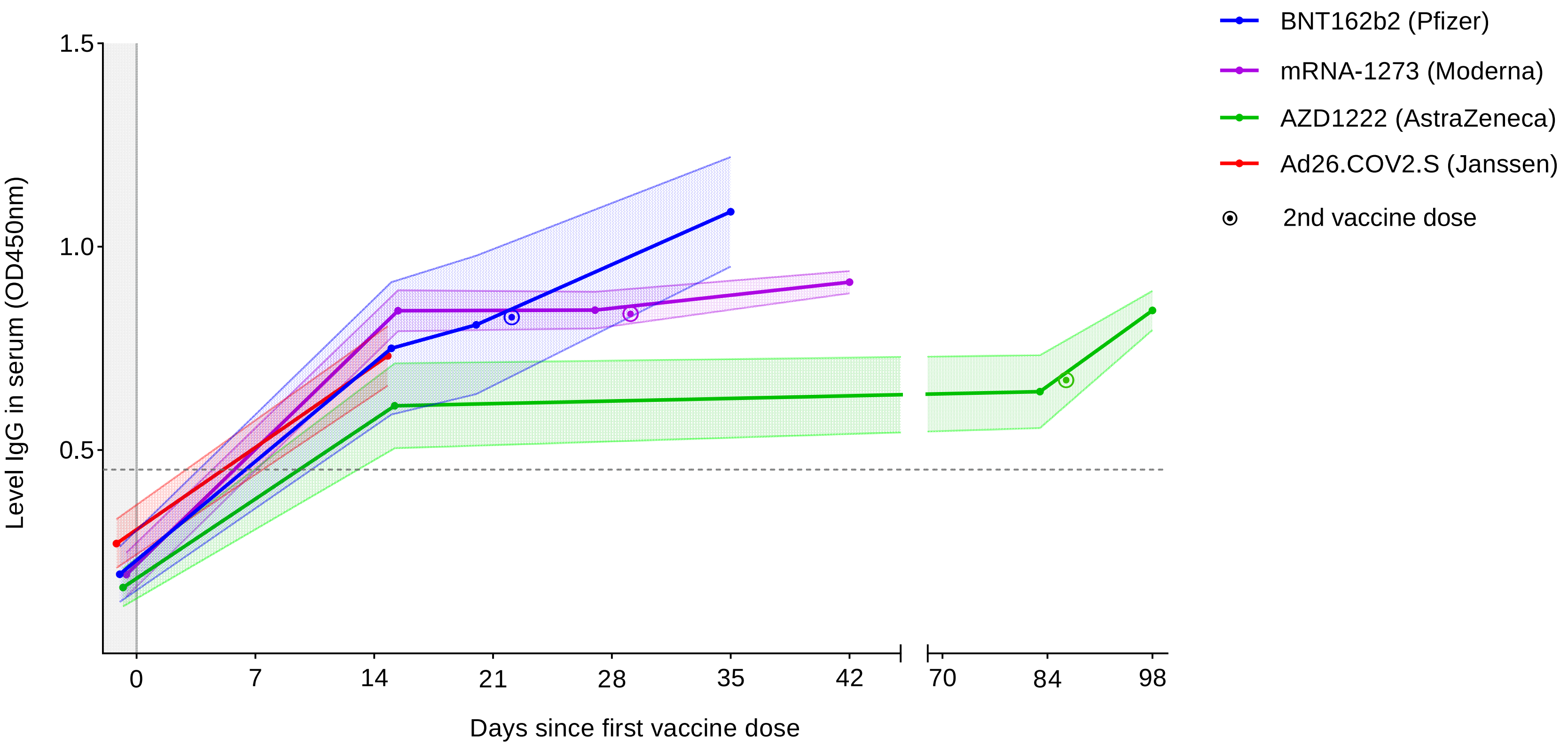

Supplemental Figure 3. Mean levels of SARS-CoV-2-specific Immunoglobulin G (IgG) in serum of all groups during entire study period.

The dotted line indicates the cut-off value for level SARS-CoV-2 specific IgG in serum; filled area between error lines indicates the standard deviation
